# Medical Society of the County of Albany

**Published:** 1871-06

**Authors:** 


BUFFALO

### Medical and Surgical Journal.

VOL. X. JUNE, 1871. No. 11.

### Original Communications.

ART. I.—*Medical Society of the County of Albany. Semi-monthly Meeting, March 14th,* 1871.

Dr. Wm. H. Bailey, President, in the Chair.

Dr. Robertson remarked that the patient whose case he had reported at the last meeting, viz: A man, æt. 29, whose kidneys were undergoing degeneration, and who was unable to see at all with one eye, and could only count the figures three feet off with the better eye, had been placed upon a milk diet alone, using it in various forms, cooked and uncooked. He had sensibly improved, the thirst, edematous condition and pasty appearance had disappeared, the sight of the better eye had improved so that he could read large type; other prospects were favorable for his recovery.

Dr. Thos. Becket, reported an interesting case of *Stricture of the Urethra,* successfully treated by himself.

Dr. James S. Bailey, reported a *Case of Spinal Lesion:*

Mr. E. R. G—, aged 43, a stout, muscular man, May 29, while carrying a heavy plank upon his shoulder a distance of three blocks, sank under its weight from syncope. He was soon restored to consciousness, but complained of pain in the lower part of his abdomen which did not yield to the application of mustard, warm fomentations, liniments, &c. He suffered without sleep until the next morning, when a physician was sent for, who prescribed an anodyne with temporary relief.

The next morning he was comparatively free from pain and rode half a mile in a lumber wagon, over a rough pavement, the jolting of which gave him much pain.

He was again compelled to send for his physician. He now could not go up and down stairs without clinging to the banisters, nor walk across the floor without assistance.

The sixth day after the accident, he was seated in his saloon; in attempting to rise to wait upon a customer, he fell upon the floor, having lost the use of his lower limbs.

For two or three days after this he could move his toes slightly. June 8th, I first saw him. He was seated in an easy chair with his legs closely wrapped in flannel blankets, complaining of their being cold; they had a peculiar doughy feel, and their sensibility was diminished; his countenance looked well, pulse normal, complete retention of urine and torpidity of his bowels, not having had a movement for three days; they readily responded to a moderate dose of castor oil, and when the catheter was introduced, one quart of higly colored urine was passed; his appetite remained good.

I ordered him placed in bed so that I could examine him more thoroughly. Upon the lower part of the spine I found a small dark spot about the size of a thumb nail, with the surrounding skin somewhat inflamed from pressure while sitting. During the day he laid on one side, and when his position was changed his hip was blistered and inflamed from the same cause. There was no tenderness along the spine except one spot over the lower part of the lumbar region, which was but slightly sensitive. There was but little change in his condition until June 12th, when his appetite became impaired, could not sleep well, skin looked dusky, sweat profusely, both hands were shriveled and cold, he was somewhat delirious, and there was numbness in his right arm, the bowels were distended with flatus, tongue coated, pulse 120, urine abundantly secreted. The discoloration over the sacrum, and inflammation of the surrounding parts extended, notwithstanding every effort to relieve the pressure.

June 14th, his legs were observed to be moist from perspiration for the first time, there was some twitching of his toes, and his legs were quite sensitive to pressure from the weight of the bedding, pulse now reduced to 100, and had occasional priapisms.

June 18.—The moving of the bedding caused spasmodic contractions of both legs, complained of an asthmatic difficulty in breathing, which was only relieved by an anodyne; turned over without assistance.

June 22.—There was but little change in his condition; tried the current of electricity, the sensation was decidedly pleasurable, and reduced the number of pulse.

June 26.—The asthmatic difficulty in breathing comes on once in 24 hours and is exceedingly annoying, passed his urine for the first time without the use of the catheter, blisters were appearing about his mouth, was very restless, which friends attribute to the extreme hot and sultry weather.

June 28.—Right foot and leg much swollen and temperature increased, left leg cold to the touch. The spasmodic difficulty in breathing lasted two hours, the blister extended down upon the neck.

July 1.—Suffered much pain, which seemed like neuralgia; there was much subsultus tendenum; countenance looks anxious; secretion of urine diminished; there is a general flagging of the system; pulse 120, small and weak, complains of feeling cold, hands cold and congested, nails blue, frequent mucous evacuations, pupils dilated.

July 3.—Pulse, p. m., 140, temperature of body 102, seems drowsy, back much more inflamed and gangrenous.

July 4.—Temperature 96, no pulse at the wrist, skin cold and clammy, pupils dilated, urine nearly suppressed, very restless, and could not get in an easy position, hands cold, shriveled and bluish; 2 p. m., pulsation of the heart very feeble, mind perfectly clear and conscious that he was dying. In half an hour he expired.

*Post Mortem*—External appearance—Rigor Mortis well marked, no emaciation, an old cicatrix of a bubo in the left groin, old cicatrices along the tibia of both legs; the left leg and thigh somewhat larger than the right; a large eschar over the right trochanter and a larger one over the sacrum.

*Thorax*—The anterior portion of the lungs were pale and healthy, considerable hyperstatio congestion at their posterior portions; slight adhesions at both apices, no tubercular deposits; the peri cardium contained no serum whatever.

*The Heart* was of normal size and appearance, but the tissues were somewhat friable; the semi lunar valves were slightly thickened.

*Abdomen*—The abdominal walls contained a thick layer of adipose tissue; the liver was large, pale and fatty; both kidneys were of normal size, but on section they presented a granular appearance, spleen slightly enlarged and soft; pancreas normal; stomach and intestines appeared healthy.

*Spinal Cord*—The spinal canal was opened anterially by removing the bodies of the vertebra; the lower dorsal and lumbar portions were removed, and considerable serous effusion was found in the theca; the vessels of the lumbar portion of the cord, as well as those of the corda equina, were very much engorged with blood; the cord was placed in alcohol for subsequent examination with the microscope, which did not reveal anything additional. Brain not examined.

This case presents many interesting features. That a man in the prime of life, physically stout and robust in appearance, should be stricken with paralysis from so trivial a cause, languish and die in a little more than one month from the accident.

It is a nice point to know the amount of lesion of the spinal cord which is sufficient to produce death. In this instance the injury seemed insufficient, yet there was complete paralysis from the hips downward, caused by overtaxing the muscular and nervous system.

The symptoms, upon first examining the case, indicated compression of the spinal cord. My opinion was subsequently confirmed by a number of the leading physicians of this city, yet no one could foretell the fatal termination. There is no doubt but the habits and excesses of the patient did much towards debilitating and hastening a fatal issue. Remedies seemed of little avail, there was a gradual wasting of the strength and tissues, although at death the body was far from being emaciated. There was a great lack of vitality in the system, as observed in the great tendency to inflammation and suppuration from pressure in the parts of the body coming in contact with the bed.

The asthmatic difficulty referred to, was undoubtedly of nervous origin, and yielded to remedies usually employed under such circumstances.

The galvanic battery was used for several days without benefit, the patient described the sensation produced by its current as agreeable. I am strongly impressed with the belief that the electric current is not applicable to acute affections of the spine, but in chronic cases I have no doubt of its utility.

For the last ten days prior to death, a careful record of the temperature of the body, twice daily, was kept, also of each of the lower extremities, together with the number of pulse before and after the use of the battery, which is given in tabular form below. The thermometer did not indicate approaching dessolution. Death took place, seemingly, from the lack of nervous stimulus to the heart. The paralysis seemed to extend up to and embrace this organ.

RECORD OF TEMPERATURE, PULSE AND RESPIRATION.

| Date. | | Time of Observation. | Body. | Right leg | Left leg | Pulse. | Resp. |
|---|---|---|---|---|---|---|---|
| June | 25 | 9 o'clock a. m. | 98° | 96° | 94° | 100 | 25 |
| " | " | After using bat. | 99° | 98° | 96° | 108 | 27 |
| " | 26 | 9 o'clock a. m. | 100° | 98° | 95° | 120 | 33 |
| " | " | After using bat. | 101° | $99\frac{2}{5}$° | $97\frac{2}{5}$° | 110 | 28 |
| " | " | 9 o'clock p. m. | 101° | $100\frac{2}{5}$° | 99° | 108 | 27 |
| " | 27 | 9 o'clock a. m. | 101° | $100\frac{3}{5}$° | 96° | 116 | 30 |
| " | " | After using bat. | 102° | 100° | 95° | 112 | 29 |
| " | " | 9 o'clock p. m. | $97\frac{3}{5}$° | $100\frac{2}{5}$° | $96\frac{2}{5}$° | 110 | 28 |
| " | 28 | 9 o'clock a. m. | $100\frac{2}{5}$° | 100° | 97° | 110 | 28 |
| " | 29 | " " " | $98\frac{2}{5}$° | $99\frac{2}{5}$° | $99\frac{2}{5}$° | 110 | 28 |
| " | 30 | " " " | $99\frac{2}{5}$° | $100\frac{3}{5}$° | $99\frac{1}{5}$° | 108 | 27 |
| July | 1 | " " " | 102° | 101° | 100° | 116 | 30 |
| " | 2 | " " " | 99° | 100° | 98° | 120 | 33 |
| " | " | " " p. m. | 102° | 102° | $99\frac{3}{5}$° | 120 | 33 |
| " | 3 | " " a. m. | 99° | 100° | 99° | 125 | 34 |
| " | " | " " p. m. | 102° | 102° | 99° | 140 | 40 |
| " | 4 | " " a. m. | 96° | 100° | 95° | No pulse at wrist | 30 |
| " | " | " " p. m. | $99\frac{1}{5}$° | 100° | 94° | Died at half past two. | |

Semi-Monthly meeting, April 24th, 1871. The President being absent, Dr. DeVoe was called to the Chair.

Dr. C. D. Mosher wished to know the experience of the members with Hydrate of Chloral. He having lost a patient by the use of one grain.

Dr. Robertson had noticed distinct conjunctivitis following its use. In doses of from 5 to 10 grains for wakefulness it was very succsssful. In his own practice had noticed a singular coldness following its use.

Dr. HUN stated that he had seen it used at the Insane Asylum at Utica in doses of 40 grains every half hour with little effect. A private patient had taken 40 grains per day for three months with no ill effect. He had never seen bad results from its use.

Dr. J. W. MOORE had found it valuable in whooping-cough.

Dr. BECKETT had a case of a woman who had been drinking excessively, and took by mistake, ʒii at one time. She slept two hours then repeated the dose. She recovered in a few hours without experiencing any ill results.

Dr. J. S. BAILEY had used it successfully in insomnia of children, had given a child one year old 5 grains with happy result.

Drs. J. S. MOSHER, NORTHROP, HALL, QUACKENBUSH and others participated in the discussion, giving their experience.

Dr. QUACKENBUSH then remarked that a quarter of a century ago he had recommended a man for membership who had been an honor to the Society by his quiet, courteous deportment, earning a large circle of friends and retaining them through years of change. As he is about to leave us for a new field of labor, he thought we owed him an expression of our good will and kind wishes for him, and would therefore offer the following. Whereas

Dr. SAMUEL H. FREEMAN is about leaving this city for the purpose of taking up his residence in the County of Saratoga, and

Whereas, he has, by an honorable conduct as a member of this society, and as one of its former Presidents sustained the honor and dignity of our profession, therefore

*Resolved,* That this society parts with regret, with its honored and esteemed associate of twenty-five years standing, and hopes that in his new abode, and from his new associates, he may receive that courtesy and kind consideration to which his urbanity of manners and his professional conduct have ever entitled him.

*Resolved,* That Dr. Freeman, in leaving this city, carries with him the good wishes and respect of every member of the Albany County Medical Society, of which he has been a member for over a quarter of a century.

*Resolved,* That this society wish Dr. Freeman continued success in his profession, and happiness in all his relations of life.

The resolutions were unanimously adopted.

Dr. MCMURDY spoke of the value of Pinus Canadensis as an astringent, and gave a sample bottle to each member. A vote of thanks followed.

Dr. J. W. MOORE then gave his experience in Turpeth Mineral in Croup.

Dr. QUACKENBUSH stated he had used it, and thought it a mechanical remedy in its action, and only to be used as a last resort. The Society then adjourned.

———:o:———

### SEMI-ANNUAL MEETING OF ALBANY COUNTY MEDICAL SOCIETY.

REPORTED BY JAMES S. BAILEY, M. D.

Dr. WM. H. BAILEY, President, in the Chair.

The minutes of the last annual meeting having been read and approved

Dr. MILTON M. LAMB, Chairmrn of the board of censors remarked that the censors had examined the credentials of the following named gentlemen, and found them satisfactory, viz:—Drs. L. R. Boyce, Lorenzo Hall, K. V. R. Lansing, Wm. H. Murray, E. B. Tefft, L. C. Graveline and Barnabas Wood. After which they were elected members.

Dr. Andrew Wilson, Vice-President, then proceeded to deliver the semi-annual address upon "The Relation of Physicians to Patients." Dr. Wilson said:

*Mr. President, and Gentlemen of the Albany County Medical Society:*

By an Act of the Legislature of this State, passed April 4th, 1806, and subsequently amended, this society was duly incorporated, and empowered to make such By-Laws and Regulations as should be deemed proper. By Article 2d of Sec. 3d of the by-laws, it is made the duty of the Vice-President to deliver before the members of the society, at the semi-annual meeting, an address "on some medical topic."

Two different constructions can be put on the words, "on some medical topic." He who would give them a strict and literal interpretation, in making his address before you, would take a single text, and devote himself to its developement, turning neither to the right nor to the left. While on the other hand, another of his brethren, regarding the words, "on some medical topic," as being vaguely directory, rather than at all mandatory, would feel free to range; and I, belonging to this latter class, shall, in offering some general desultory remarks, feel conscience clear as to the discharge of the duty of addressing you.

It was said of the Great Physician, "He went about doing good." There can be no higher eulogium than is contained in that simple sentence, and it is one of the peculiar rewards of our exacting and most trying profession, gentlemen, that it bestows on each capable and conscientious member the comfort and consolation of those words, "He went about doing good." I speak of capable and conscientious members, for alas! in our profession, as in that of law and divinity, there is the sham, the quack, the empiric, all "seeking whom they may devour." What the quack is, is just what the physician ought not to be; and what the physician is, that the quack cannot be, from the nature of the case. The empiric, in having one medicine for all diseases, has, in reality, a reliable medicine for none. His greedy ambition continually overleaps itself. The true physician, on the other hand, in humility and faith, works out the cause of some of the ills to which the flesh is heir, and meets them with effectual remedies. To the success of the former, ignorance and great professions of skill are essential, while to the latter, knowledge, patience, and a realization of the wisdom of the words, "becoming fools that they may be wise."

Diseases are so many and so complicated, patients so differ with their perplexing peculiarities, that in important cases no one feels the necessity of a correct diagnosis, and the importance of a correct medicine as demanded by that diagnosis, more than does the physician. Hence in consultations, while there is nothing so offensive to the physician as the flippant arrogance of the quack, he does not feel that he has done his whole duty to the patient or exhausted his resources, until he has availed himself of the best consultation he can procure. Want of means is not a valid excuse for the neglect of his duty. Very often a suggestion from a thoughtful and intelligent fellow-worker may induce the correction of errors and an adoption of treatment that is saving. We all know how common and useful in ordinary life is the suggestion of a friend of culture and experience. The spreading out before him of your difficulty is a common benefit; both become wiser therefrom. In the medical profession, as elsewhere, "friend sharpeneth friend." It is true that in the ultimate resort, every practitioner must rely on his own judgment, but human life must not be sacrificed to an obstinate adherence to an unsuccessful method of treating a case.

Perhaps there is no more delicate duty than the duty of consultation. The necessities of the patient, the realization of what is due to oneself, either in the character of regular attending, or special consulting physician, as well as what is due to anxious friends of the patient ; all this presents one of the difficulties and trials of our profession in a most emphatic manner. Life and friendship may hang on a single suggestion, even upon a word, aye , even upon the manner of expressing either.

We have thus spoken of the physician in *contradistinction* from the empiric, contrasting the true with the false, the real with the counterfeit; and have dwelt for a moment on the difficulties incident to a wise discharge of the delicate, difficult, yet most important duty of consultation. And yet, delicate and difficult as is this last mentioned duty, it would be less grevious to be borne, if it could be left to physician with physician. Not that I would, by any means, undervalue the suggestions of the intelligent and experienced nurse, who, with that peculiar "suaviter in mode," the especial prerogative of woman, in such a valued assistant to every doctor. By no means, to the faithful nurse, can be fitly applied the beautiful words of Scott:—

> "O woman! in our hours of ease,
> Uncertain, coy, and hard to please,
> And variable as the shade
> By the light quivering aspen made ;
> When pain and anguish wring the brow,
> A ministering angel thou !"

But while I thus would pay due honor to the regular nurse, I will make war against those attendants at the sick-room who are neither nurse nor physicians, and are always pregnant with opinions and suggestions. I allude to those individuals whom Cooper in the "Spy" calls "the bitch doctors of the regiment." There are those who worry the family, disturb the sick room, and presume to show the nurse how every duty is to be discharged. These unconsulted consulting physicians, can inform all who will listen to them, with an absolute certainty, what will cure the patient; the fact that they are ignorant of the disease not making the slightest difference in any case. Some universal quack has given them a universal remedy, and they regard it as potent and able to save in all cases. They see no reason why it should not check a raging fever, or drive off malignant small-pox, for, look you, it once cured a sprained ankle, and was known to have been the salvation of a child troubled with worms!

An incident that occurred in my own practice is apposite just here. When I was just starting in my professional life, I had as a patient, one of the most prominent and influential citizens of the place in which I was located. He was a very sick man, and the entire village, as well as his immediate friends and family, were very anxious and alarmed about him. I tell you, gentlemen of this society, I felt most deeply the responsibilities of my profession. It was in the dead of winter, and the residence of my patient was away among the cold bleak hills of Duansburgh, where they look off upon the beautiful valley of the Mohawk as it stretches towards Schenectady. I made my way through snow drifts across the fields as best I could, until I reached one of the main roads running through the valley to the city. I induced a professional brother to accompany me to the chamber of our sick friend, and upon reaching our destination, we were at once surrounded by a bevy of ex-officio unconsulted consulting old women, each of whom was sure she could mention a specific for the cure of our patient, without so much as knowing what ailed him. The good old doctor whom I had brought with me, listened to their clatter in silence for a few minutes, and then looking around at his helpers, remarked with great good humor, and yet with a show of solemnity and sternness, "Won't you all be silent, I think I know as much about this case *as you and I put together*." The speech did not tend to the doctor's popularity among his auditors, but his directions were followed, and the patient recovered.

We have thus far spoken of doctors in their professional relations. I now propose, in conclusion, to say a word or two as to their intercourse with one another. The phrase, "when doctors disagree who shall decide," implies that sometimes even the members of our profession fail to see eye to eye. How can it be otherwise, living as we doctors do, in a world in which there are no two things alike? Did the geologist ever find two stones exactly alike? Or the botanist two flowers? Or the astronomer two stars? Did the student of humanity ever find two men alike in every particular? Did any member of our profession ever have a patient exactly duplicated? There is but one answer to all these questions, and that is an undisputed negative.. Medicine, no more than Divinity, is an "exact science," and no man is fit to be a doctor unless he thinks for himself, and who supposes that thinking men will all arrive at identical conclusions? It cannot be! and it must happen, therefore, that doctors will disagree.

It is no disparagement to doctors of medicine, law or divinity, to be bold and *independent thinkers*, and being so, their *differences* are to their *credit* as showing energy and vigor of mind. This topic could be treated of beyond the limit proper on this occasion, so I shall content myself with the few words I have already uttered in regard to it. I should, however, hold myself *derelict* in duty if I did not, at the *expense* of inclination, make a single remark relating to unfriendly differings in the profession. He has read the Inspired Word to very little purpose, where it dwells on man's intercourse with his fellow-man, who has forgotten the injunction so pregnant with wisdom, "First cast out the beam out of thine own eye, and then shalt thou see clearly to cast the mote out of thy brother's eye." It is the violation of this teaching of the plainest common sense, this most charitable precept of the great "Sermon on the Mount," that leads to undue and most unwise *asperity* in difference. Both parties to the controversy are *presented* to the world in an unfavorable light, *irrespective* and disregardless of their professional standing, of which outsiders are too often not competent judges.

I cannot close this part of my subject better than by presenting a quotation from an authority none here can question:—

"Honor and Justice," he writes, "particularly forbid a medical practitioner's infringing upon the rights and privileges of another who is legally accredited, and whose character is not impeached by public opinion or civil or medical authority; whether he is a native or a stranger settled in this country. There is no difference between physicians but such as results from their personal talents medical acquirements, or their experience, and the public, from the services they receive, are the natural judges of their intellectual advantages. In all probability, every good physician would receive a merited share of patronage, were there not many who usurp a portion through artful insinuation and slander of others, or combination against, or improper interference with the more worthy practitioner. Any physician thus molested or injured, is justifiable in applying for redress to the County Medical Society to which he is attached."

I have thus, gentlemen, presented you some random, disconnected thoughts, on some general topics connected with our profession, a profession to which, I believe, we are all devotedly attached. We love it, not so much, in my humble opinion, for the gain that attaches to its successful prosecution, as because of its own mate sake. Medicine, like law and literature is "its own exceeding great reward." The physician, if he be worthy of the name, if he is studious, conscientious and patient, is one of the noblest of God's creations; he is a philanthropist in one of the best senses of that much abused term. Day by day, fervent prayers from anxious hearts accompany him as he goes in and out in his professional rounds. The most important earthly issues are committed to his hands, life itself depending constantly on his decisions, as controlled by Providence. The profession is full of trials, but it is likewise full of triumphs; and, in conclusion, gentlemen, thanking you for the kind attention with which you have honored me, permit me to express the hope that for each and every one of you, the trials of the coming years may seem as nothing, because of the overweight of triumph. And saying this, I ask you to join with me e'er I sit down, in the wish that our Albany Society may have before it many years of usefulness, and be in the future as in the past, the scene of many interchanges of friendly greetings, and the medium of much that is of great professional value.

